# How Much Vancomycin Dose Is Enough For The MRSA Infection in Pediatric Patients With Various Degrees of Renal Function?

**DOI:** 10.22037/ijpr.2019.1100654

**Published:** 2019

**Authors:** Yang Wang, Ping Gao, Huanian Zhang, Yujun Chen, Changhe Niu, Maochang Liu, Sichan Li, Qiong Xu, Qi Ye

**Affiliations:** 1 Department of Clinical Pharmacology, Wuhan Medical & Healthcare Center for Women and Children, Hubei, China.; 2 Y W. P G. contribute equally to this article.

**Keywords:** vancomycin, Children, Population Pharmacokinetics, Monte Carlo Simulation, Drug resistance, Nephrotoxicity

## Abstract

Today, an increase in vancomycin dose has been proposed to ensure efficacy. However, the risk of nephrotoxicity will increase with the dose. The aim of this study was to evaluate the dosage regimens of vancomycin in pediatric patients based on pharmacokinetics/pharmacodynamics (PK/PD) and to optimize dosage individualization. Population pharmacokinetics analysis was performed on 155 Chinese children (aged 1 month to 16 years), which were divided into various renal function subpopulations. Monte Carlo simulation was carried to evaluate the efficacy and safety of vancomycin dosage regimens on each subpopulation. Compared with children with normal renal function as glomerular filtration rate (GFR) ≥ 90 mL/min·1.73 m^2^, the clearance of vancomycin decreased by 39.4% and the half life increased 1.74 fold respectively in children with moderate renal inadequacy (30 ≤ GFR < 60 mL/min·1.73 m^2^). When vancomycin was administered as conventional dosage (40-60mg·kg^-1^·d^-1^) to against methicillin-resistant staphylococcus aureus (MRSA) with higher MICs of 1-2 mg·L^-1^ for children with normal renal function, the probability of efficacy target attainment ( PTA) at AUC_0-24h_/MIC ≥ 400 (where AUC is the area under curve and MIC is the minimum inhibitory concentration) achieved ≤ 63.64%. While vancomycin dosage exceeded 70mg·kg^-1^·d^-1^ for children with normal renal function, 50mg·kg^-1^·d^-1^ for mild renal inadequacy (60 ≤ GFR < 90 mL/min·1.73 m^2^), 30 mg·kg^-1^·d^-1^ for moderate renal inadequacy respectively, the PTA at trough concentration above 20 mg·L^-1^ achieved > 20%, that not to be suggested for high risk of nephrotoxicity. Considering both efficacy and safety, the conventional vancomycin dosage is not enough and adjustable interval is narrow for pediatric patients with MIC 1-2 mg·L^-1 ^MRSA infection and normal renal function.

## Introduction

Vancomycin, a member of the glycopeptide antibiotics, was isolated from *Streptomyces orientalis* in 1957. It is still the first choice for treating methicillin-resistant Staphylococcus aureus (MRSA), methicillin-resistant Staphylococcus epidermidis (MRSE) and other Methicillin Resistant Coagulase-Negative Staphylococci (MRCNS) (1). With the increase of vancomycin-intermediate and vancomycin-resistant Staphylococcus aureus (VISA and VRSA) (2, 3), it has been widely recognized that individual use of vancomycin should be based on the pharmacokinetic / pharmacodynamic (PK / PD) theory. Vancomycin is mainly excreted by the kidneys, so its pharmacokinetic profile was significantly affected by the renal function of patients (4). Compared to adults, pharmacokinetic parameters of vancomycin in the pediatric population, neonates and infants in particular, were inﬂuenced by growth and maturational changes in renal function (5, 6). Children with different renal function are likely to have large interindividual variability in vancomycin pharmacokinetics and in whom the dosage guidelines are not clearly established. Meanwhile, it has been demonstrated that tissue penetration of vancomycin and antibacterial effect against MRSA were closely related with vancomycin trough concentration (C^s^_min_) and AUC_0-24h_/MIC (where AUC_0-24h_ is the area under the drug concentration-time curve in a 24-h interval and MIC is the minimum inhibitory concentration). 

Therefore, to ensure efficacy C^s^_min _and AUC_0-24h_/MIC were suggested to maintain at 10-20 mg·L^-1^ and ≥ 400, respectively (4-11). These parameters were established in adult patients and extrapolated in pediatric patients with MRSA infection (6). However, the renal function of most of the children increased compared with adults, and thus would be expected to be even less likely to achieve AUC_0-24h_/MIC ≥ 400 in serious MRSA infection (5). It is unknown whether the conventional vancomycin dosing for children with different renal function can result in an AUC_0-24h_/MIC ≥ 400. In addition, vancomycin may lead to severe concentration-dependent ototoxicity and nephrotoxicity (12). For these reasons, individualized medication is necessary for vancomycin to ensure the best clinical outcome and minimum risk, especially for geriatrics and pediatric patients. However, vancomycin pharmacokinetics in Asian children, Chinese children in particular, was rarely reported, thus restricting its individualized use. Besides, the efficacy and safety of conventional regimen needs further proof when applied into patients with different renal function. In the present study, we established a population pharmacokinetics (PPK) model in Chinese children at the first step. And then the correlation between vancomycin regimen and its clinical efficacy as well as its safety were evaluated under different bacterial resistance and renal function by using Monte Carlo Simulation (MCS) to provide a reference for vancomycin individualized therapy.

## Experimental

*Patients and Treatment protocol*

All patients admitted from March 2009 to January 2014 in Wuhan Medical & Healthcare Center for Women and Children, Hubei, China. Patients were eligible for our study if they were diagnosed with severe pneumonia, septic arthritis, bacteremia or bacterial meningitis with MRS (including MRSA, MRSE and the other MRCNS); showed no obvious improvement of clinical signs after initiating therapy with the other antibiotics over 72 h, and drug sensitive test showed the pathogens were sensitive to vancomycin; were strict compliance with vancomycin therapy. Patients who meet the following standard were excluded from this study. whose key data were missing; whose sampling time was not clear or wrong; received treatment which may affect the vancomycin pharmacokinetics such as prolonged intermittent renal replacement therapy or high-volume haemofiltration (13, 14). Vancomycin (Eli Lilly Japan K.K, Seishin Laboratories) was given by intravenous infusion and its dosage or frequency were in line with the package insert or adjusted according to the clinical manifestation (Table 1).

The study protocol was approved by the Medical Ethics Committee of Wuhan Medical & Healthcare Center for Women and Children. The legal representative or guardian of each participant provided written informed consent before entering the study in compliance with the applicable local regulations.

*Sample Collection and analysis*

1 to 3 blood samples about 3 mL for vancomycin concentration determination were drawn from a peripheral vein of each patient after the fourth dose, and the time between dosing and sampling was recorded. Blood samples were centrifuged and the serum was obtained; all of the serum samples were kept frozen at -20 ℃ until the analysis was taken.

For vancomycin analysis, 1 mL serum was loaded onto a solid phase extraction column, and eluted with 10% acetonitrile. Serum samples were analyzed for vancomycin with Agilent high performance liquid chromatography (HPLC) system and a Hypersil C_18_ column (4.6mm×200mm, 10 μm). The mobile phase consisted of acetonitrile- potassium dihydrogen phosphate (9:91) and the flow speed was 0.8 mL/min. The detecting wavelength was 236 nm.

 The linear range of vancomycin in serum was 0.9-117.3 mg/L. 

The limits of detection (LOD) was 0.4 mg/L; The mean recovery of vancomycin in QC samples at concentrations of 0.9, 59.1 and 117.3mg/L were between 96.7%~101.7%, both of the intra-day and inter-day relative standard deviation (RSD) were less than 5%. 

*Determination of biochemistry and MIC*

The biochemical analysis including alanine aminotransferase (ALT), aspartate aminotransferase (AST), direct bilirubin (DBIL), total bilirubin (TBIL), urea nitrogen (BUN), and creatinine (CRE) were determined using Cobas 8000 (Roche Diagnostics Ltd, Forrenstrasse, Switzerland). Glomerular filtration rate (GFR) was calculated by the modified Schwartz’s formula (15). 

The MIC values were determined using the standardized agar dilution method. If more than 1 bacterium was isolated, the highest value was used as the defnitive MIC. 

*Data Analysis*

*Software and hardware*

PPK software: Phoenix® NLME program (version 1.2, Pharsight Corporation, USA); MCS software: Oracle® Crystal Ball software (version 11.1.2.1.0, Oracle Corporation, USA); Computer: Dell precision® desktop computer (version T5500, Dell Inc. China) with the Windows 7 operating system.

*Population PK model development*

*Structural model*

The population PK analysis was performed using a non-linear mixed-effect model approach implemented using NLME computer program, a population pharmacokinetic model was ﬁtted to vancomycin serum concentrations versus time data from a total of 351 samples obtained from 155 patients aged 1 month to 16 years. The ﬁrst order conditional estimation method with the η-ε interaction option (FOCE L-B) was used to obtain parameter estimates. Based on previous modeling performed on other studies (16, 17) and exploratory graphical analysis, one- and two-compartment structural kinetic models with first-order elimination were evaluated. The best structural model was chosen based on an examination of the objective function value (OBJ, equal to the -2 log-likelihood value of the data) and the visual inspection of standard goodness-of-fit plots, including the individual fits. 

*Error model*

The exponential model was used to describe the inter-individual variability of the PK parameters as shown in Equation 1:

$P_{i}=\mathrm{\theta .Exp}(n_{i})$

Equ.1

Where P_i_ is the pharmacokinetic parameter for the i^th^ individual, θ is the population typical value of the according parameter, and η_i_ is a random variable for the i^th^ individual following a Gaussian distribution with a mean of 0 and a variance of ω^2^.

The intra-individual variability (residual error) was evaluated using an addition model according to

 Equ. 2 $$C_{\mathrm{obs}}=C_{\mathrm{pred}}+\varepsilon$$

Where C_obs_ is the observed serum vancomycin concentration, C_pred_ is the corresponding model predicted concentration, and ε is assumed to follow a Gaussian distribution with a mean of 0 and a variance of σ^2^.

*Covariate analyses*

Demographic variables (including gender, age, height, weight, and body surface area), hepatic and renal functions (including ALT, AST, DBIL, TBIL, BUN, CRE, GFR) of patients were tested as potential covariates for the PK parameters. Categorical covariates were incorporated using indicator variables with an exponential function. The influences of continuous covariates were included in the model using a power function. Covariates were chosen using stepwise forward selection-backward elimination with the likelihood ratio test. Because the OBJ follows a chi-square distribution, a covariate was considered significant when the addition of the covariate resulted in a decrease in the OBJ (ΔOBJ) of greater than 3.84 (P < 0.05; df = 1), and the elimination of the covariate resulted in an increase in the OBJ (ΔOBJ) of greater than 6.63 (P < 0.01; df = 1). After a full covariate regressive model was established, the analysis procedure was continued until only significant covariates remained in the final model.

*Model evaluation*

The PPK final model was evaluated using Bootstrapping, Visual Predictive Check (VPC) and goodness-of-fit plots, including observations against population or individual predictions scatterplot, distribution of residuals. Using the final model. 1,000 bootstrap-resampled data sets from the original model group data sets were sequentially estimated. The median and 95% confidence intervals (95% CIs) (2.5th percentile and 97.5th percentile) of the parameters obtained from this step were compared with the final parameter estimates. A VPC was carried out for the ﬁnal model for which 1,000 datasets were simulated. 

*Groups and MCS*

According to the grade standard for renal function recommended by National Kidney Foundation (NKF-K/DOQI, 2002) (18), 155 patients were divided into three groups: normal (GFR ≥ 90 mL/min/1.73 m^2^), mild renal insufficiency (GFR 60-89 mL/min/1.73 m^2^), and moderate renal insufficiency (GFR 30-59 mL/min/1.73 m^2^). Patient with GFR <30 mL/min·1.73 m^2^ or renal failure were not involved. After fixing the PK parameters and variation in the final model, the PK parameters for individuals in three groups of different renal function were obtained by using post hoc empirical Bayesian approach. The mean values and standard deviation of the parameters in different groups were calculated. Based on the previous reports (5-10, 19-22) and guidelines (4, 11), AUC_0-24h_/MIC was chosen as the indicator of vancomycin efficacy against MRSA and AUC_0-24h_/MIC ≥ 400 was considered effective. Vancomycin trough concentration (C^s^_min _) were regarded as the indicator of safety and C^s^_min_ > 20 mg·L^-1^ indicated high risk of nephrotoxicity. Pharmacokinetic parameter was assumed following the lognormal distribution, dosage regimen, MIC and renal function of patients were setup, and then Monte Carlo simulations run for 10 000 times according to the formula (1) and (2). 

Probability of target attainment (PTA) for the indicators was used to evaluate the efficacy and safety of various dosage regimens under preset scenes.

$\frac{{\mathrm{AUC}}_{0-24h}}{\mathrm{MIC}}=\frac{\mathrm{Dose}}{\mathrm{CL}∙\mathrm{MIC}}$

Equ. 3

$C_{\min}^{\mathrm{ss}}=\frac{x_{0}}{k_{e}V_{d}T}(e^{k_{e}T}-1)(\frac{1}{1-e^{-k_{e}t}})e^{-k_{e}t}$

 Equ. 4

Where dose is vancomycin daily dose, CL is clearance, k_e _is elimination rate constant, V_d _is apparent volume of distribution, x_0 _is single dose, T is the infusion time, τ is dosing intervals.

*Statistical analyses*

Descriptive statistics were expressed as the arithmetic mean ± SD unless otherwise specified. The statistical analysis was carried out using SPSS Statistics for Windows software (version 13.0) by Student’s t-test and one-way analysis of variance followed by post hoc test. P < 0.05 was considered significant. 

## Results

*Patient characteristics*

**Figure 1.**
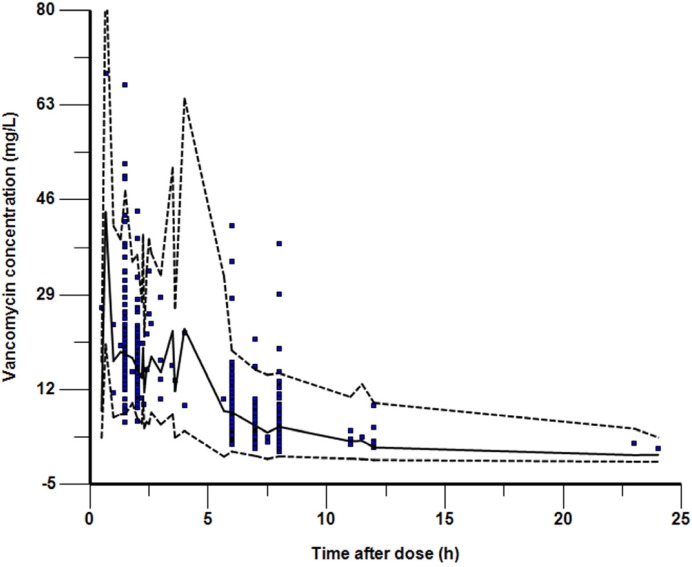
Visual predictive check for ﬁnal model showing observed Vancomycin serum concentrations. The solid line shows the 50th percentile of the simulated data and the dashed lines show the 2.5th and 97.5th percentiles. The visual predictive check plots obtained after stratiﬁcation by weight demonstrated a similar level of concordance between observed and simulated data

In total, 155 patients ranging in age from 1 month to 16 years (110 patients were younger than 2 years, accounted for 71%) with 351 samples were enrolled into this study. The demographic and clinical characteristics of the patients were summarized in Table 1. The intervals between the last dosage time and sampling time were distributed over 0.5–24 h (Figure 1).

**Table 1:** Baseline characteristics of patients for population pharmacokinetics modeling (n = 155, mean ± SD)

**Characteristics**	**Value**
Patient Data	
Gender (male:female)	65:90
Age (months)	25.86±35.22
Height (cm)	79.21±25.43
Weight (kg)	10.94±6.86
Body surface area (m2)	0.48±0.23
Laboratory parameter	
ALT (IU·L-1)	46.01±92.51
AST (IU·L-1)	70.35±104.03
DBIL (µmol·L-1)	4.58±6.41
TBIL (µmol·L-1)	11.19±16.85
BUN (mmol·L-1)	3.48±1.86
CRE (µmol·L-1)	33.72±17.34
GFR (mL/min·1.73m2)	138.84±69.32
Vancomycin dosage regimen	
Single dose(mg·kg-1)	14.42±3.90
Daily dose (mg·kg-1·d-1)	40.57±8.84
Dosing interval time(hr)	8-24

Glomerular filtration rate (GFR) was calculated with modified Schwartz formula.

**Table 2:** Hypothesis test results of covariates affecting on vancomycin clearance (CL)

**Hypothesis test**	**OBJ**	**ΔOBJ**	**P-value**	**Comments**
Basic model	2206.3			
If Gender affect CL ?	2205.0	-1.3	>0.05	NO
If Age affect CL ?	2097.6	-108.7	<0.01	YES
If Weight affect CL ?	2094.0	-112.3	<0.01	YES
If Body surface area affect CL ?	2095.4	-110.9	<0.01	YES
If ALT affect CL ?	2204.8	-1.5	>0.05	NO
If AST affect CL ?	2204.8	-1.5	>0.05	NO
If CRE affect CL ?	2197.5	-8.8	<0.01	YES
If BUN affect CL ?	2205.6	-0.7	>0.05	NO
If GFR affect CL ?	2140.6	-65.7	<0.01	YES

**Table 3:** Full model development process and statistical analysis

**Step**	**Model structure**	**OBJ**	**ΔOBJ**	**P-value**	**Comments**
0	Vd=θVd . Exp(ηVd); CL=θCL . Exp(ηCL)	2206.3			Basic model
1	Vd=θVd . Weight^θWeight . Exp(ηVd); CL=θCL . Exp(ηCL)	2074.6	-131.7	< 0.01	
2	Vd=θVd . Weight^θWeight . Exp(ηVd); CL=θCL . Weight^θWeight . Exp(ηCL)	1959.2	-115.4	< 0.01	
3	Vd=θVd . Weight^θWeight . Exp(ηVd); CL=θCL . Weight^θWeight . lnGFR^θlnGFR . Exp(ηCL)	1927.3	-31.9	< 0.01	
4	Vd=θVd . Weight^θWeight . Exp(θGender) . Exp(ηVd); CL=θCL . Weight^θWeight . lnGFR^θlnGFR . Exp(ηCL)	1922.5	-4.8	< 0.05	Full model

θWeight, θlnGFR and θGender were the fxed parameters relating to covariates; ΔOBJ was the change of objective function value while adding

or deleting one covariate from prior model.

**Table 4:** Parameter estimates (standard errors, SE) and Bootstrap results for the ﬁnal model

**Parameter**	**Final model estimate (SE)**	**Bootstrap estimate**	
		**Median**	**95%CI**
θVd (L) (typical value for Weight=9 kg)	5.33(0.75)	5.39	3.528~7.252
θ (L·h-1) (typical value for Weight=9 kg and CL GFR=121.5 mL/min·1.73m2)	1.08(0.54)	0.98	0.401~2.574
Exponent for Weight as covariate for Vd	1.027(0.063)	1.018	0.858~1.183
Exponent for Weight as covariate for CL	0.858(0.083)	0.852	0.686~1.022
Exponent for lnGFR as covariate for CL	2.367(0.430)	2.378	1.503~3.253
ωVd(%)	21.4(2.5)	21.3	15.5~27.1
ωCL(%)	24.4(2.9)	24.4	16.9~31.9
σ(mg·L-1)	0.52(0.052)	0.51	0.238~0.630

**Table 5:** Pharmacokinetic parameters of subpopulation groups with various renal function status estimated with Bayesian method (n=155, mean ± SD)

**Group**	**N**	**CL(L·kg** **-1** **·h** **-1** **)**		**V** **d ** **(L·kg** **-1** **)**		**k** **e** **(h** **-1** **)**	
		**Estimate**	**95%CI**	**Estimate**	**95%CI**	**Estimate**	**95%CI**
GFR ≥ 90	111	0.142±0.059	0.131-0.153	0.65±0.27	0.60-0.70	0.238±0.090	0.221-0.255
60 ≤ GFR < 90	24	0.131±0.091	0.092-0.169	0.68±0.43	0.50-0.86	0.195±0.072^a^	0.164-0.225
30 ≤ GFR < 60	20	0.086±0.043^a^^b^	0.066-0.106	0.66±0.29	0.53-0.80	0.137±0.062^a^^b^	0.107-0.166
Total	155	0.133±0.066	0.123-0.144	0.65±0.30	0.61-0.70	0.218±0.091	0.204-0.233
P value		0.002		0.861		0.000	

Compared with GFR ≥ 90 mL/min·1.73m2 subgroup:

a *P *< 0.05, compared with 60 ≤ GFR < 90 mL/min·1.73m2 subgroup:

b *P *< 0.05, using one-way analysis of variance followed by post hoc test.

**Table 6:** Simulation results of AUC0-24h/MIC of vancomycin for various dosage regimens under various preset scenes (including bacterial drug resistance and patient renal function).*

**MIC of bacteria**	**Dosing regimen**	**AUC** **0-24h** **/MIC**, **mean (≥ 400 probability/%)**		
		**GFR ≥ 90**	**60 ≤ GFR **< **90**	**30 ≤ GFR **< **60**
0.25 mg·L-1	10mg·kg-1·d-1	330.1(25.00)	459.6(45.84)	559.5(70.65)
	20mg·kg-1·d-1	657.8(85.71)	913.7(83.35)	1138.8(98.23)
	30mg·kg-1·d-1	995.0(98.30)	1367.1(94.78)	1701.2(99.85)
	40mg·kg-1·d-1	1322.7(99.74)	1825.8(98.17)	2249.5(100)
0.50 mg·L-1	20mg·kg-1·d-1	329.6(24.66)	456.5(46.18)	565.4(71.45)
	30mg·kg-1·d-1	495.7(63.05)	692.8(70.46)	847.6(93.30)
	40mg·kg-1·d-1	659.0(85.31)	915.1(83.96)	1130.6(98.23)
	50mg·kg-1·d-1	828.1(94.50)	1132.9(90.73)	1409.7(99.57)
0.75 mg·L-1	30mg·kg-1·d-1	329.8(24.54)	451.4(45.38)	562.8(70.97)
	40mg·kg-1·d-1	441.1(51.53)	605.6(63.64)	757.0(89.02)
	50mg·kg-1·d-1	552.8(72.70)	760.7(76.01)	940.9(95.98)
	60mg·kg-1·d-1	662.8(85.12)	919.4(83.60)	1131.3(98.40)
1.00 mg·L-1	30mg·kg-1·d-1	245.6(7.31)	340.2(28.48)	419.6(45.34)
	40mg·kg-1·d-1	327.0(23.96)	454.1(44.76)	561.9(71.41)
	50mg·kg-1·d-1	413.7(45.85)	569.8(58.93)	702.1(85.84)
	60mg·kg-1·d-1	495.6(63.64)	688.1(70.25)	845.7(92.75)
1.25 mg·L-1	30mg·kg-1·d-1	196.9(2.45)	272.9(17.92)	339.3(27.88)
	40mg·kg-1·d-1	263.1(10.52)	363.0(31.98)	452.7(52.34)
	50mg·kg-1·d-1	330.0(24.44)	458.1(46.05)	561.7(70.94)
	60mg·kg-1·d-1	395.6(41.09)	545.9(57.55)	677.4(83.40)
1.50 mg·L-1	30mg·kg-1·d-1	165.7(0.93)	229.9(11.97)	283.3(16.02)
	40mg·kg-1·d-1	220.7(4.54)	303.9(22.48)	379.5(37.14)
	50mg·kg-1·d-1	275.5(12.92)	372.7(34.00)	469.6(55.86)
	60mg·kg-1·d-1	330.8(25.00)	459.6(46.18)	562.5(70.68)
1.75 mg·L-1	30mg·kg-1·d-1	141.6(0.24)	197.9(7.69)	243.0(9.12)
	40mg·kg-1·d-1	188.3(1.77)	264.5(16.74)	323.2(24.35)
	50mg·kg-1·d-1	236.3(6.34)	324.3(25.49)	403.6(41.80)
	60mg·kg-1·d-1	283.1(14.21)	393.4(37.02)	483.5(58.22)
2.00 mg·L-1	40mg·kg-1·d-1	166.1(0.76)	229.8(12.10)	283.3(15.87)
	50mg·kg-1·d-1	206.8(3.23)	283.5(19.11)	350.8(30.38)
	60mg·kg-1·d-1	248.0(8.15)	343.1(28.66)	424.8(46.94)
	70mg·kg-1·d-1	287.0(15.01)	399.4(37.27)	493.5(59.78)

A total of 10 000 virtual pediatric patients were simulated at each regimen.

**Table 7:** Simulated vancomycin trough concentrations at steady state for various dosage regimens to virtual patients with various renal function status

**Dosing regimen**	**Prediction of C** **s ** **, mean (> 20 mg·L** **-1 ** **probability/%)** **min**		
	**GFR ≥ 90**	**60 ≤ GFR < 90**	**30 ≤ GFR < 60**
10mg·kg-1·d-1, tid, 1-h infusion	1.59(0.00)	2.57(0.18)	4.30(0.50)
20mg·kg-1·d-1, tid, 1-h infusion	3.28(0.17)	5.14(1.88)	8.65(6.96)
30mg·kg-1·d-1, tid, 1-h infusion	4.93(1.09)	7.63(5.74)	12.72(17.5)
30mg·kg-1·d-1, tid, 2-h infusion	5.42(1.58)	8.41(7.14)	13.81(19.42)
40mg·kg-1·d-1, tid, 1-h infusion	6.58(3.40)	10.31(11.37)	17.28(30.10)
40mg·kg-1·d-1, tid, 2-h infusion	7.21(3.87)	11.22(13.54)	18.08(32.20)
50mg·kg-1·d-1, tid, 1-h infusion	8.18(6.44)	12.94(17.88)	21.62(40.97)
50mg·kg-1·d-1, tid, 2-h infusion	9.17(8.29)	13.95(20.53)	22.59(44.00)
60mg·kg-1·d-1, tid, 1-h infusion	9.69(9.73)	15.66(24.62)	25.57(50.11)
60mg·kg-1·d-1, tid, 2-h infusion	10.82(12.34)	16.89(27.92)	27.15(53.64)
70mg·kg-1·d-1, tid, 1-h infusion	11.58(14.54)	17.95(29.94)	30.19(59.00)
70mg·kg-1·d-1, tid, 2-h infusion	12.73(17.61)	19.38(34.17)	31.26(61.13)
80mg·kg-1·d-1, tid, 1-h infusion	13.22(19.24)	20.80(35.83)	34.47(65.05)
80mg·kg-1·d-1, tid, 2-h infusion	14.55(22.70)	22.38(40.26)	36.32(67.56)

A total of 10 000 virtual pediatric patients were simulated at each regimen.

**Figure 2:**
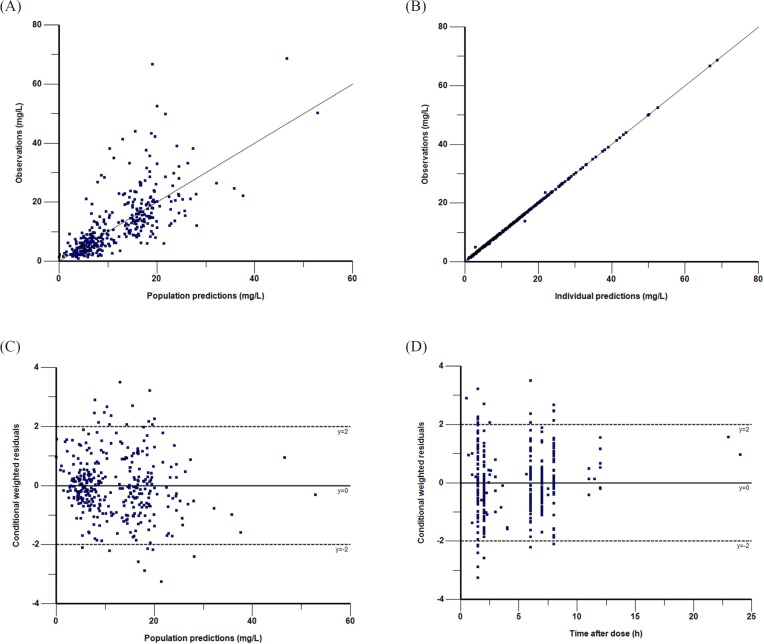
Goodness-of-fit plots of the final PK model in modeling group. Observations against population (A) or individual (B) predictions. The line of identity is shown. Conditional weighted residuals against population predictions (C) or time after dose (D). The line where conditional weighted residuals are equal to 0 is shown

**Figure 3:**
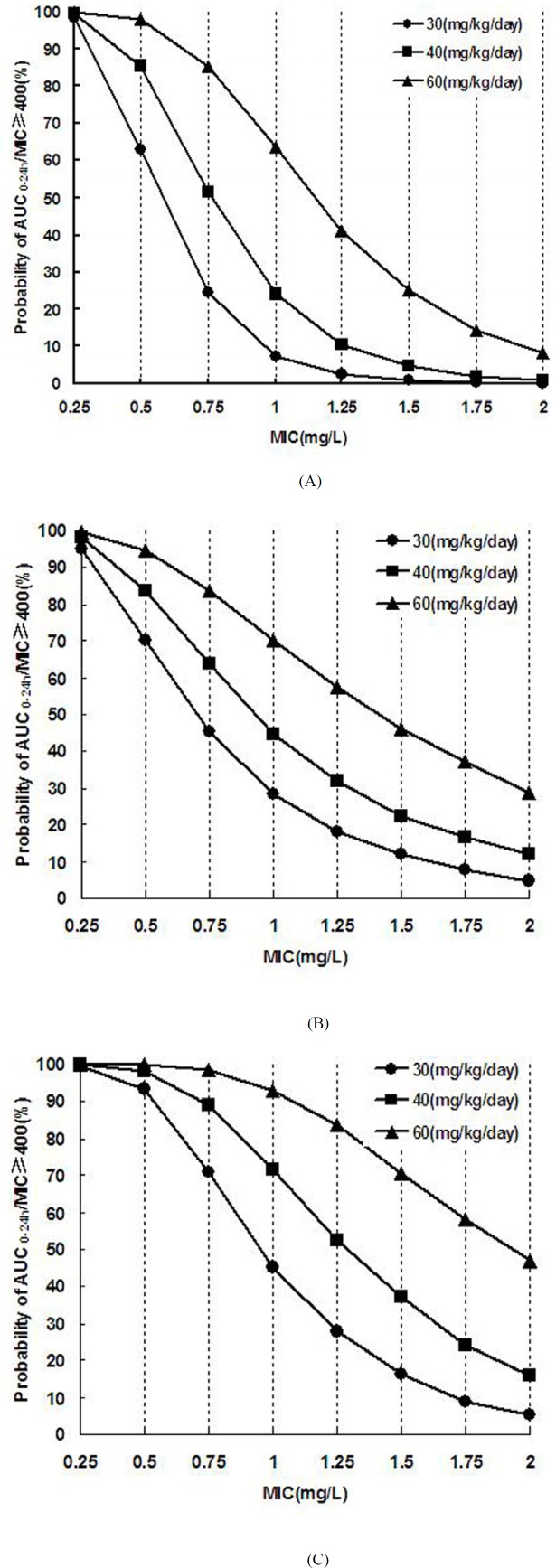
Probabilities of achieving AUC0-24h/MIC ≥ 400 target for various dosage regimens of vancomycin and various MICs.

(A) The GFR ≥ 90 mL/min·1.73m2 subgroup. (B) The 60 ≤ GFR < 90 mL/min·1.73m2 subgroup. (C) The 30 ≤ GFR < 60 mL/min·1.73m2 subgroup.

**Figure 4:**
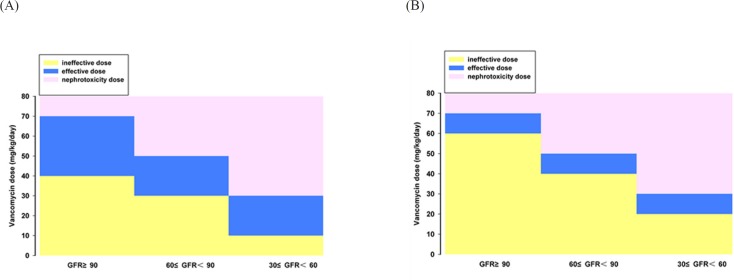
The dose range of vancomycin for children with different renal function. (A) for bacteria with MIC < 1 mg·L-1. (B) for bacteria with MIC 1-2 mg·L-1

*Population Pharmacokinetics*

A one-compartment, open kinetic model with first-order elimination best described the data. Parameterisation of the ﬁnal one compart-ment model was in terms of systemic clearance (CL) and apparent volume of distribution (V_d_). This model resulted in OBJ drops of 78.6 from the comparable two-compartment model. The hypothesis test results of covariates affecting on vancomycin CL were summarized in Table 2. A summary of the full model development process is shown in Table 3 in decreasing order of the OBJ (model improvement).

In the final model, the equations by which to derive the population values for CL, V_d_ are as follows: 

θ_Vd_ (L) = 0.558×Weight^1.027^

θ_CL_(L·h^-1^)=0.004×Weight^0.858^×(lnGFR)^ 2.367^.

Where Weight is given in kg, and GFR is given in mL/min·1.73m^2^.

The parameter estimates and bootstrap conﬁdence intervals for the final model are shown in Table 4. The typical population values of the pharmacokinetic parameters estimated in final model after normalization to the median weight of 9 kg and median GFR of 121.5 mL/min·1.73 m^2^ in these subpopulations were as follows:The V_d_ was 5.33 L; the CL was 1.08 L·h^-1^ and the corresponding half-life estimate was 3.4 h. The population estimates for the final model were very similar to the median of the bootstrap replicates (relative error was within ± 10%) and within the 95% confidence interval obtained from the bootstrap analysis. VPC of the ﬁnal model indicated that the model provided a reliable description of the observed data (Figure 1). The goodness-of-fit plots in Figure 2 show that the model predictions were in reasonable agreement with the observed plasma concentrations. No trend in the conditional weighted residuals versus the time-after-dose plots was observed. Most of the conditional weighted residuals were evenly distributed around zero and within an SD of ±2 of the normalized units.

*Individual pharmacokinetic parameters*

The pharmacokinetic parameters for individuals were calculated by Bayesian analysis (Table 5). Vancomycin clearance and elimination rate constant showed significant differences in the three groups (P < 0.05), indicating renal function played a critical role in vancomycin clearance. Compared with the group with normal renal function, the clearance decreased by 39.4% and elimination rate constant decreased by 42.4% (or the half life increased 1.74 fold) in patients with moderate renal insufficiency. 

*Evaluation of the efficacy*

Bacterial resistance and renal function of patients under various dosage regimens were setup in advance, and then Monte Carlo simulations run for 10 000 times according to the formula (1) and the PK parameters in Table 3. The mean value and PTA of AUC_0-24h_/MIC were obtained to evaluate the efficacy of different dosage regimens under preset scenes (Table 6). Higher PTA for AUC_0-24h_/MIC ≥ 400 suggested better expected efficacy. As showed in Table 6, when patients with normal renal function (GFR ≥ 90 mL/min·1.73m^2^) were given vancomycin 40 mg·kg^-1^·d^-1^ to kill the bacteria with MIC ≤ 0.50 mg·L^-1^, the PTA of AUC_0-24h_/MIC ≥ 400 was above 85.31% in the 10 000 simulation tests, indicating a high efficiency against infection. However, with the improvement of bacterial resistance (MIC ≥ 0.75 mg·L^-1^), the success rate of conventional dose 40mg·kg^-1^·d^-1^ was less than 51.53%, suggesting a poor expected efficacy. When the MIC reached to 1-2 mg·L^-1^, even given a high dose of 60mg·kg^-1^·d^-1^, the efficiency against infection for patients with normal renal function were below 63.64%. For patients with mild renal insufficiency, the dose of 40mg·kg^-1^·d^-1^ can achieve a high success rate (PTA ≥ 83.96%) against the bacteria with MIC ≤ 0.50 mg·L^-1^. 

For patients with moderate renal insufficiency, the dose of 30mg·kg^-1^·d^-1^ can achieve good efficiency (PTA ≥ 93.3%) for bacteria with MIC ≤ 0.50 mg·L^-1^. The PTA of AUC_0-24h_/MIC ≥ 400 versus MIC under different vancomycin dosage regimens were showed in Figure 3. With the increase of MIC, the success rate decreased, whereas failure happened in certain conditions.

*Evaluation of the risk*

Assuming that patients with different renal function were given various vancomycin dosing regimens, Monte Carlo simulations run for 10 000 times according to the formula (2) and corresponding PK parameters. The C^s^_min_ and PTA of C^s^_min_ > 20 mg·L^-1^ were obtained to evaluate the risk of nephrotoxicity for different vancomycin regimens (Table 7). As showed in the table, the PTA of C^s^_min_ > 20 mg·L^-1^ occurred when the patients with normal renal function, mild or moderate renal insufficiency received vancomycin more than 70 mg·kg^-1^·d^-1^, 50 mg·kg^-1^·d^-1^, or 30 mg·kg^-1^·d^-1^, respectively, which suggested the risk of nephrotoxicity is higher than 20%. Hence, the above dose regimens should not be recommended to the corresponding populations. If the excess dose is necessary, clinician should weigh the risks and benefits and strengthen the monitoring of concentration.

## Discussion

With the globally increase of the bacterial resistance, it has been recognized that individual use of antibiotics should be based on the PK/PD principle. Personalized medication is useful to improve efficacy, decrease resistance and minimize toxicity, especially for the drugs with severe adverse reaction (e.g., vancomycin), or population with larger variability on pharmacokinetics (e.g., geriatrics and pediatrics). FDA is advocating the use of computer simulation technology to develop clinical dosage regimen of antibiotics, design assumptions and research the effect of variables on the results, prediction and evaluation of potential benefits and risks of different regimens, in order to provide objective evidence to develop therapeutic schedule. This technique can effectively avoid the dilemmas in real clinical trial such as limited sample size and large subjective interference. MCS is an analytical method for random events or "experiments" based on artificial creation, usually obtains the probability of occurrence of a specified goal by running thousands of simulations, in order to provide reference for decision-making. Most of the published reports on antibiotic regimen optimization by using MCS were based on the PK data of healthy volunteers. Due to the difference between healthy subjects and patients, the applicability of MCS results was limited. MCS research based on the PPK is the focus of academic field in the present, which can truly reflect the disposition of drugs under clinical disease states. 

In this study, the data of 155 children (aged from 1 month to 16 years) infected with MRS were used to establish a PPK model suitable for Chinese children. And then the PK parameters were obtained by Bayesian method. The mean values of CL and V_d_ were 0.133 ± 0.066 L·kg^-1^·h^-1^ and 0.65 ± 0.30 L·kg^-1^, respectively. Our result were similar with that of Wrishko *et al*.(16) (in which CL= 0.12 L·kg^-1^·h^-1^ and V_d_= 0.57 L·kg^-1^) and Yasuhara *et al*.(17) (in which CL = 0.119 L·kg^-1^·h^-1^ and V_d_= 0.522 L·kg^-1^). But the value of CL is faster than that reported by Marques-Minana *et al*.(23) (0.066 L·kg^-1^·h^-1^) in neonates, probably due to that, the age of the children in the present study were older. About 80%~90% of vancomycin was excreted by glomerular filtration in less than 24 h (8), some by renal tubular secretion (24), therefore renal function, especially the glomerular filtration rate (GFR) will directly affect the *in-vivo* elimination rate of vancomycin. Dailly *et al*. (25) found a positive correlation between GFR and vancomycin clearance in 70 patients suffered burns. Chen YC *et al*.(26) found the correlation coefficient between GFR and vancomycin clearance was 84.65% in 65 patients infected with MRSA. Many vancomycin PPK studies also found various covariates which indirectly reflect the GFR such as CRE(27), Ccr (28), or cysteine proteinase inhibitor in serum c (Cystatin c) (26, 29) were significant correlated with vancomycin clearance. Modified Schwartz′s formula (15) was used to calculate GFR in children with this study and it was successfully integrated into the final model, well elaborated the quantitative relationship between GFR and vancomycin clearance. After grouping according to NKF-K/DOQI standard, we found vancomycin clearance and elimination rate constant in children with 30 ≤ GFR<60 mL/min·1.73m^2^ decreased by 39.4% and 42.4% compared with the children with GFR ≥ 90 mL/min·1.73m^2^, which proved once again the necessary of adjustment for vancomycin dosage. 

After establishing the vancomycin PPK model and obtaining the PK parameters in patients with different renal function, a large sample (10 000 cases) simulation "experiment" was conducted by using MCS to explore the efficacy and safety of various dosage regimen under different preset scenes. The correlation between vancomycin antimicrobial effect against MRSA and AUC_0-24h_/MIC has been reported since 90′s of the last century in animal models (7, 8). Subsequently, a number of large samples of human PK/PD studies (5, 9, 10) confirmed that vancomycin organizational penetration and anti-infection is optimal when its AUC_0-24h_/MIC was above 400. Current United States infectious disease society (IDSA) (4, 11) and Chinese expert consensus recommends that AUC_0-24h_/MIC ≥ 400 should be regarded as the target indicator. Our MCS results indicated that the dose of 40mg·kg^-1^·d^-1^ was effective against MRSA strains with MIC ≤ 0.50 mg·L^-1^ (PTA of AUC_0-24h_/MIC ≥ 400 was ≥ 85.31%) in patients with normal renal function. But this dosage regime may fail to kill the bacteria with MIC ≥ 0.75 mg·L^-1^ (PTA ≤ 51.53). In that case, a larger dose was needed. For MRSA infection with MIC 1-2 mg·L^-1^, the success rate is not enough (63.64%) even given a dose of 60mg·kg^-1^·d^-1^. In 2006, United States Clinical Laboratory Standardization Association (CLSI) reduced the susceptibility breakpoint of vancomycin from 4 to 2 mg·L^-1 ^(11, 30), whereas our simulation results indicated that the conventional doses of 40-60mg·kg^-1^·d^-1^ is only effective for MRSA with MIC ≤ 1 mg·L^-1^ but inadequate for MRSA with MIC 1-2 mg·L^-1^. Eiland *et al*. (31) analyzed the vancomycin TDM results of 295 children and found vancomycin given at 40-60mg·kg^-1^·d^-1^ was difficult to achieve effective target trough concentration (10-20mg·L^-1^). They calculated using a formula and concluded that 70mg·kg^-1^·d^-1^ was needed for patients with normal renal function to achieve the target trough concentration of 10 mg·L^-1^. Giachetto *et al*. (32) also observed that the probability of AUC_0-24h_/MIC ≥ 400 is only 50% even given vancomycin 39.92mg·kg^-1^·d^-1^ for MRSA strains with MIC 1 mg·L^-1^. Frymoyer *et al*. (33) suggested that the probability of AUC_0-24h _> 400 will be over 90% when vancomycin trough concentration was at least 10 mg·L^-1^. That is, vancomycin trough concentration may not need to reach 10 mg·L^-1^ for MRSA strains with MIC ≤ 1 mg·L^-1^, whereas for MRSA strains with MIC 1-2 mg·L^-1^ the concentration should be more than 10 mg·L^-1^ which was difficult to achieve routine dose. Therefore, supernormal dosage is permissible when the bacterial resistance is increased in order to ensure clinical efficacy. However, the risk of adverse drug reaction will increase with the dose. Even though the ototoxicity is rarely reported after the purity of vancomycin was improved, many toxicologic studies have demonstrated that vancomycin nephrotoxicity sharply increased when its trough concentration was above 20 mg·L^-1 ^(19-22), especially for co-administration with nephrotoxic drugs such as aminoglycoside (4). Our simulation results indicated that the probability of C^s^_min_ > 20 mg·L^-1 ^will be more than 20% when vancomycin dose is 70,50, and 30 mg·kg^-1^·d^-1^ respective for patients with GFR ≥ 90 mL/min·1.73 m^2^, 60 ≤ GFR < 90 mL/min·1.73 m^2^ and 30 ≤ GFR < 60 mL/min·1.73 m^2^. In that cases, the probability of nephrotoxic were greater, so the corresponding dose was not recommended. Combined with the simulation results of efficacy and safety, the reasonable dose range of vancomycin for children with various degrees of renal function is obtained (Figure 4). We concluded that the range of vancomycin dose is narrow for MRSA strains with MIC 1-2 mg·L^-1^; hence, the treatment will be more difficult and the cure rate is not high. Since the MRSA strains with MIC 1-2 mg·L^-1 ^are prone to mutate into heterogeneous Vancomycin-Intermediate Staphylococcus aureus (hVISA), producing offspring MIC ≥ 4 mg·L^-1^ resistant subpopulation (30), which should be paid attention and a better way to fight infection was necessary. For this type of strain, the clinicians can increase the dose of vancomycin under blood concentrations monitoring and toxicity monitoring, or combine vancomycin with rifampicin (34) or fosfomycin (35), or replace with lower nephrotoxic drugs such as linezolid, daptomycin, and teicoplanin for the patients with moderate or even worse renal function.

The present study suggested that individualized dosing regimen of vancomycin should be developed in accordance with bacterial resistance and renal function of the patients, and fully considering the pros and cons in order to ensure the efficacy and safety of antibiotic therapy. According to the simulation results, 40-70, 30-50, 10-30 mg·kg-1 vancomycin daily dose for MIC ≤ 1 mg·L-1 MRSA infection treatment is adapted to pediatric patients with GFR ≥ 90, 60 ≤ GFR < 90 and 30 ≤ GFR < 60 mL/min·1.73m^2^ , respectively. And the adjustable dosage interval of vancomycin is narrow for treating MIC 1-2 mg·L^-1 ^MRSA infection. The limitation of the study is the number of the patients with renal insufficiency is small, so fitting errors may exist for the pharmacokinetic characteristics of such population. We will collect more patients in the future to in-depth explore the regularity of vancomycin PK/PD in Asian children. 

*Compliance with Ethical Standards*

Funding: This work was funded by The Key Project of Science and Technology of Wuhan (2013060602010258) and The National Futang Fund for Children’s Medicine Development (2015 No.33).

Conflicts of interest: All authors have no potential conflicts of interest to declare.

Ethical approval: All procedures in this study were carried out in accordance with the 1964 Helsinki declaration and its amendments and the ethical committee or institutional review board which approved the study.

Informed consent: Written informed consent was obtained from the legal representative or guardian of each patient when they began treatment but was not required for the present analyses because this was a retrospective study.
